# Supplementary material for: Are carbon emissions trading and green financial instruments synergistic? -Comprehensive quantitative research based on content analysis
Source: PLoS One. 2024 Mar 7;19(3):e0298601. doi: 10.1371/journal.pone.0298601 (PMC10919601; doi:10.1371/journal.pone.0298601)
Supplement: S1 Data — (ZIP) [file pone.0298601.s001.zip › Data/地级市Z_F工作报告.zip!/╜¡╦╒/╤∩╓▌╩╨/└┤╫╘╣1⁄2╓┌║┼í╛┬φ┐╦╩2╛▌═°í┐_2016.docx]

各位代表：

现在，我代表扬州市人民政府向大会报告工作，请予审议，并请市政协各位委员和其他列席人员提出意见。

2015年和“十二五”时期工作回顾

2015年，面对复杂的宏观经济环境和艰巨的改革发展任务，我们在中共扬州市委领导下，认真贯彻党的十八大和十八届三中、四中、五中全会精神，以习近平总书记系列重要讲话和对江苏工作最新要求为根本遵循，把握和适应新常态，克难求进，开拓创新，全市经济社会保持平稳健康发展势头。预计实现地区生产总值4080亿元，增长10.2%；一般公共预算收入336.8亿元，增长14.1%，其中税收占比81.6%；城镇、农村常住居民人均可支配收入分别增长9%、9.5%。节能减排实现省定目标。

1.坚持主动作为，加快转型升级，经济运行稳中有进

项目建设成效明显。新落户阿尔斯通高压母线管等世界500强及跨国公司项目5个，新增省认定跨国公司地区总部2家。省级重大项目比上年增加5个。新开工宝应日升铜合金等工业重大项目50个。近三年竣工的65个项目实现开票销售、入库税收分别占规上工业的18%和25%。重大科技成果转化项目居全省第二。150个亿元以上技改项目完成投资191.2亿元。新开工万达广场等服务业重大项目42个、竣工运营项目29个。新增规上工业企业156家、规上服务业企业217家。

实体经济平稳增长。围绕增信心、稳增长，召开系列产业发展推进会和工业企业家千人大会，出台“服务企业50条”政策措施。工业万元开票销售入库税收增长14.4%。用活2亿元企业应急专项资金。国税和地税为企业减、免、退税115亿元。帮助企业直接融资180亿元，“新三板”挂牌企业达35家。恒丰银行扬州分行开业。新增私营企业2.1万户、个体工商户3.9万户。新增中国驰名商标6件、省著名商标49件。

三次产业加快转型。五大千亿级产业、高新技术产业、战略性新兴产业产值分别占规上工业的69.1%、44.5%、29.4%。新能源产业出口交货值增长1.5倍。节能环保产业产值增长10.5%。工业机器人保有量增长30%。建筑业总产值3100亿元，增长12%。新创鲁班奖2项。建设综合性游客服务中心10个。高邮盂城驿创成国家4A级景区。广陵信息服务产业基地成为江苏首个省市共建“互联网产业园”，市经济技术开发区“扬州智谷”获批省大数据特色产业园。软件和信息服务业收入增长40%以上。服务业增加值占GDP比重43.5%，增长贡献率首次超过工业。粮食生产实现“十二连丰”。农业基本现代化稳步推进，6个50亿元农业特色产业基地基本建成。

三大需求拉动有力。全年完成固定资产投资2856亿元，增长18.2%。工业技改投资增长27.2%，高新技术产业投资增长10.8%。服务业完成投资1330亿元，增长24.2%。全市社会消费品零售总额1237亿元，增长9.7%。实现电商交易额320亿元，增长40%。市区商品房销售276万平方米、成交总额234亿元，分别增长16.6%、15.7%。进出口总额增长3.3%。境外投资项目22个，其中“一带一路”项目9个。

2.突出创新引领，强化统筹协调，发展活力不断增强

科技创新能力增强。实施“科教合作新长征”和“科技产业合作远征”计划，签订产学研合作协议669项，落户校企研创中心56家。出台支持科技服务业发展意见，科技服务业收入增长15%。制（修）定国家标准12项、行业标准5项。国际标准化组织饲料机械技术委员会秘书处落户扬州。发明专利授权量增长60%。新增国家高新技术企业71家，获省高新技术产品认定838项。新增省级以上“两站三中心”42家。引进高层次领军人才107名、产业急需的专业技术人才1010名。

重点领域改革深入推进。推进行政审批制度改革，实施企业注册登记“三证合一”和“一照一码”工作。在省内率先完成行业协会商会与行政机关脱钩。实施市级行政事业单位所属企业清理规范和全面监管，市属监管企业资产总额同比增长18.2%。启动新一轮综合医改，城市公立医院全面实行药品零差率销售。新组建卫生与计划生育委员会。完成全市市场监管体制改革。实施食品药品监管体制改革。完成300个村土地承包经营权确权颁证。农村集体资产股份合作制、农村产权交易市场建设实现新突破。仪征被列为全国农民住房财产权抵押贷款试点。

功能载体优化升级。扬州高新区晋升国家高新区。省级科技产业园实现县（市、区）全覆盖。15家工业集中区创成市级特色产业园。城区商业综合体完成投资38.9亿元。22个科技产业综合体投入使用产业用房190.5万平方米，入驻企业741家。众创空间入驻创业企业345家。建立机械装备、软件信息、综合检测3个联合创新中心。江苏省玉器产品质检中心顺利建成。高邮八桥获批国家农业科技园区，江都获批国家级现代农业示范区，广陵食品产业园通过国家农业产业化示范基地认定。

区域发展协调推进。《扬州市城市总体规划（2011-2020年）》获国务院批准。我市成为长三角健康服务业专业委员会牵头城市。宁扬城际等10个项目成为南京都市圈重大基础设施合作项目。全市园区落户上海转移项目20个，

总投资301亿元。文昌路西延、新万福路及万福大桥建成通车，仪征、江都到主城更加便捷。市区三区社会保险和民政福利实现“同城同步同标”。

3.提升功能品质，放大城庆效应，名城特质不断彰显

重大基础设施建设实现新突破。连淮扬镇铁路扬州段全线开工，宁启铁路复线电气化改造全面完成。扬州泰州机场获批一类航空口岸并开通5条国际（地区）航线。352省道江都段、宿扬和江广高速加快建设。西部交通客运枢纽建成启用。城市南部快速通道、金湾路、611省道沿湖大道、芒稻船闸扩建工程、界首运河大桥等开工建设。高邮运东船闸扩建工程建成通航，长江六圩弯道应急护岸工程、淮河入江水道整治主体工程完工。

城市功能和管理水平不断完善。围绕建城2500周年，按序时推进100项城庆重大城建项目和30项政府主导城建项目，市民中心和科技馆、廖家沟中央公园主体工程、新体育场、李宁体育园等项目建成或运营。实施头桥水厂扩建工程，完成六圩污水处理厂三期工程。建成全市域桥梁信息管理系统与市区地下管线信息系统。着力推进文明城市建设长效化管理，开展农贸市场、交通秩序等7个专项整治，拆违拆破拆烂309处，在文明城市省级考评中名列前茅。创成省首批优秀管理城市。

新农村建设加快推进。编制完成《新型城镇化与城乡发展一体化规划》。新改建农村公路328公里、桥梁98座。疏浚县乡河道202条，整治村庄河塘5031个。完成农村危房改造3091户，实施渔民上岸安居850户。农村无害化卫生户厕基本普及。新创省级绿化示范村87个、“优美乡村”10个。全面完成人均年纯收入低于5000元人口的脱贫任务。宝应获批全省农业开发高标准农田建设整县推进试点县，争取省以上资金5.66亿元。农机、粮食、供销、气象在“三农”工作中发挥重要作用。

生态文明建设取得成效。编制完成《扬州市生态文明建设规划》。落实市人大《关于切实加强全市水环境保护和大气污染防治的决议》。推进市区黑臭河道治理。完成28项淮河流域水污染防治工程。整治淘汰小型燃煤锅炉509台。全面取缔古城和景区核心区露天烧烤。对市区92个工地、635辆渣土车安装监控设备。PM2.5年平均浓度比2013年下降21.4%。严控农业面源污染。实施节能改造项目101项、循环经济项目22项。启动10大生态中心和城市公园体系建设，全市成片造林3.1万亩，市区新增绿地127.5万平方米。获批国家循环经济示范城市。创成省国土资源节约集约模范市、省大气污染防治工作优秀城市。

4.优化公共服务，加强社会建设，人民生活水平日益提高

社会事业全面进步。所有县（市、区）创成国家级义务教育基本均衡县。新改建公办幼儿园6所。热点高中实现70%指标定向分配。职校招生专业订单培养达72%。有序推进分级诊疗。组建苏北人民医院医疗集团。18家农村区域性医疗卫生中心开工建设。“四位一体”全民阅读服务体系列入全国示范项目。市文化馆获全国优秀文化馆评分第一。扬州电视台区域收视份额全国第一。486非遗集聚区建成开放。成功举办“烟花三月”国际经贸旅游节。承办江苏大运河旅游推广月和中外丝路城市美食文化交流活动。完成第一次全国可移动文物普查。隋炀帝墓遗址公园景观一期工程完工。南河下片区入选国家首批历史文化街区。《扬州市志（1988-2005）》编纂发行。圆满举办市第十二届运动会暨“爱祖国、爱家乡”群众歌咏大会。获第十九届省运会承办权。人口计生、科普、台湾事务、侨务、外事和工会、青年、妇女、儿童、老龄、慈善等事业取得新进步。

民生实事工程扎实推进。加快建设“不淹不涝”城市，整治城市积水点8个。完成扬州闸改建等“清水活水”重点工程。改造区域供水支管网742公里。新辟、优化公交线路42条，新开通主城区到高邮、仪征、江都公交线路3条，购置新能源公交车370辆。新增公共自行车2500辆、租赁服务点97个，覆盖面积175平方公里。标准化体育休闲公园实现县（市、区）全覆盖。农村健身设施提档升级工程提前完成。全市新增公积金缴存职工7.7万人，新建棚户区改造安置房13489套。市区改造“城中村”15处，整治老小区82万平方米，新增老旧小区基本物业服务116万平方米，新建（筹集）公租房1436套，解决超腾仓期未安置住房13429套。完成10个农贸市场新改建任务。

居民收入和社会保障水平稳步提高。新增城镇就业7.1万人、农村劳动力转移就业1.6万人，城乡劳动者职业技能培训4.1万人。居民社会养老保险基础养老金最低月标准上调至105元。职工基本医保统筹基金支付限额由3万元调整为6万元、大病医疗救助基金由25万元提高至30万元。制定实施《医养融合发展三年行动计划》。为最年长2500名老人和五保老人发放城庆礼包，提高90周岁以上老年人尊老金标准。贫困精神病人免费用药救助标准从600元上调至3000元。我市成为全国“救急难”社会救助综合试点单位，急难家庭救助基金实现全覆盖。

社会治理体系不断健全。立体化治安防控体系取得初步成效。平安建设、综合治理列全省第一方阵。乡镇（街道）全面推行“政社互动”。进一步规范信访事项复查复核工作。“积分选岗、阳光就业”退役士兵安置模式获全省推广。积极应对和处置各类突发事件。扎实推进安全生产标准化、事故隐患排查治理和重大危险源防控，事故起数和死亡人数保持“双下降”。开展抗战胜利70周年纪念系列活动。促进军民融合深度发展。“扬州舰”光荣入列。顺利通过第七次全国双拥模范城考核验收。民族、宗教、民防、地震、仲裁等工作扎实开展。

5.坚持依法履职，推进公正公开，法治政府建设成效显著

作风建设持续深化。扎实推进“三严三实”专题教育，持续开展“三下三联三交”活动。学习贯彻《中国共产党廉洁自律准则》和《中国共产党纪律处分条例》。严格落实党风廉政建设责任制，严厉惩治腐败行为。重点抓好审计突出问题整改。围绕药品销售许可等制定“三直接”十大环节操作规范。整合建成扬州市公共资源交易中心。举办政风热线省市联动直播活动。

依法行政不断强化。自觉接受市人大及其常委会的法律监督、工作监督和市政协的民主监督，广泛听取各民主党派、工商联、无党派人士和社会各界意见。办理市人大代表议案、建议388件，政协提案395件，其中市长领办建议、提案11件。办理行政复议案件385件。制定深入推进依法行政加快建设法治政府的实施意见，取得地方立法权并出台市政府规章制定程序暂行规定。制定市若干重大行政行为程序规定。组建市政府法律顾问工作办公室，构建政府法律顾问工作体系。

政务公开有力推进。推行行政审批事项等“五个清单”制度，清理并公布部门责任清单。加强政府新闻发布制度建设。全面推进行政权力网上公开透明运行，在运行平台上共办理行政权力事项2238.7万件。推进市级部门财政预决算和“三公”经费公开，全市“三公”经费支出减少10.3%。公车改革按时完成。

政府效能得到提升。全面深化“三减一提”行政效能提升工作。市政务服务中心新址投入启用。实施政务服务系统首问首办负责制、服务承诺制、实时评价制等。为65个事项开辟绿色通道，有效缩短项目审批时限。按照国务院督查落实年总体部署，完善督查机制，确保事事有着落、件件有结果。12345政府服务热线和《寄语市长》回复率均超过96%、满意率88%以上。

各位代表，2015年工作任务的完成，标志着“十二五”发展顺利收官，为率先全面建成小康社会奠定了坚实基础。

过去的五年，是扬州重大项目建设持续发力，综合实力实现新跨越的五年。全市GDP突破4000亿元，是2010年的1.8倍，列全国百强城市第42位，比2010年前移14位；人均GDP超过14000美元，在苏中苏北地区率先超省均。一般公共预算收入是2010年的2倍，固定资产投资是2010年的2.1倍。三次产业结构由7.2:55.7:37.1调整为5.9:50.6:43.5。累计新开工工业、服务业重大项目367个、新竣工投产210个，分别是“十一五”的5.9倍和6倍。沿江100亿元、沿河50亿元重大项目实现两轮全覆盖。

过去的五年，是扬州跨江融合发展聚焦推进，转型升级取得新成效的五年。以“跨江融合发展综合改革试点”为总抓手，推进重点领域和关键环节改革，制定实施168项改革任务。以开放促发展，新增世界500强企业项目38个，进出口总量突破百亿美元，外经营业额年均增长20%，开发园区对全市经济贡献率达48.4%。实施创新驱动战略，高新技术产业产值实现翻番，科技进步贡献率达60%，人才总量比2010年增长56%。五次蝉联“全国科技进步先进市”称号，荣获国家创新型试点城市。

过去的五年，是扬州现代发展格局基本形成，区位优势得到新提升的五年。主动融入长三角，积极推进宁镇扬同城化。加快建设现代立体交通体系，扬州泰州机场、瘦西湖隧道、江六高速、新淮江公路等建成运营，连淮扬镇铁路等取得突破。高速公路、一级公路里程分别比“十一五”末增长19.2%、78%。城建工程数量、体量和投资额超过前十年之和。顺利实施市区部分行政区划调整，中心城区面积由309平方公里扩大至640平方公里，“一体两翼”、“一核多组团”发展布局进一步优化。

过去的五年，是扬州生态宜居特色不断放大，城乡统筹取得新进展的五年。按照建设“古代文化与现代文明交相辉映的名城”目标，持续改善城市功能和品质，先后创成国家森林城市、全国文明城市、国家生态市，牵头大运河申遗取得成功。建成区绿化覆盖率达43.6%。统筹沿江沿河发展，分别制定专项行动计划，宝应、高邮获省苏中苏北结合部专项扶持政策。加快推进新型城镇化和城乡一体化，实施“美好城乡建设行动”，9个乡镇入选全国重点镇，全市城镇化率达62%。

过去的五年，是扬州城乡居民获得更多实惠，民生改善再上新台阶的五年。每年将新增财力70%用于民生支出。连续11年按总体10%标准上调企业退休人员基本养老金。围绕群众基本需求，实施区域供水、“八老”改造、“1161菜篮子”、公交优先、被征地农民转保等工程，城乡居民收入分别比2010年末增长71.6%和82.4%。全市文博场馆建设超过百家，高考普通类本二上线人数连续三年突破万人，实现国家卫生城市“四连冠”，创成江苏省体育强市，公共安全感连续多年位居全省前列。

五年来，我们始终坚持同心同向、苦干实干，一着不让抓落实。紧扣市委决策部署、紧扣发展第一要务、紧扣为民富民靠民，心往一处想，劲往一处使，务实干事，合力成事。始终坚持保持定力、精准发力，聚焦聚力打好“组合拳”。围绕稳增长，出台服务企业“2号文件”、人才“6+1”等系列政策举措；围绕调结构，协同推进支柱产业高新化、新兴产业规模化、传统产业特色化；围绕惠民生，把“1号文件”作为首要任务，说到做到，做成做好。始终坚持改革创新、调适关系，不断释放发展的内生动力。正确处理政府有形之手与市场无形之手关系，积极用好内外两个市场、两种资源，充分调动市和县（市、区）多个积极性，让一切有利于创新创造的活力竞相迸发。始终坚持法治思维、法治方式，制度化推进各项工作。政府工作强化建章立制，服务发展注重简政放权，城市建设规范“四明确两评估”，确保行政权力运行依法依规、全程留痕。始终坚持问题导向、结果导向，不断加大科学发展、协调发展力度。紧盯发展后劲培育、区位交通改善、城市功能优化等关键点，抓住城乡统筹、机会均等、扶贫救困中的薄弱环节，注重扩优势、补短板，疏堵点、通节点，持之以恒，久久为功。始终坚持谋在远处、干在实处，不断推进经济社会发展迈上新台阶。以生态文明建设、居民收入增加、更有质量效益的增长为倒逼，努力在更高层次上提升城市的综合竞争力和可持续发展能力。

各位代表！“十二五”时期我市经济社会发展所取得的成就来之不易，所取得的经验弥足珍贵。这既是全市上下认真贯彻党中央、国务院和省委、省政府正确决策的结果，也是全市上下开拓创新、踏实奋斗、团结拼搏的结果。在此，我谨代表扬州市人民政府，向全市人民，向人大代表、政协委员，向驻扬单位、各垂直管理部门和所有关心、支持、参与扬州建设和发展的社会各界人士，致以崇高的敬意和衷心的感谢！

在肯定成绩的同时，我们也清醒地看到，全市经济社会发展中还存在一些问题和挑战。主要是：经济下行压力加大，内需不旺与外需不足并存，部分企业生产经营困难；转型升级任务艰巨，经济国际化程度还不高，生态文明建设仍需加强；沿江沿河之间、市区县域之间发展仍不平衡，基本公共服务能力和均等化水平与群众期望还有不小差距；社会治理能力有待进一步提高，政府依法行政、职能转变、反腐倡廉仍需加强，等等。这些问题需要我们高度重视并切实加以解决。

“十三五”时期的规划构想

各位代表，根据《中共扬州市委关于制定扬州市国民经济和社会发展第十三个五年规划的建议》，市政府制定了“十三五”规划纲要（草案），提请本次大会审议。

“十三五”时期扬州经济社会发展的指导思想是：高举中国特色社会主义伟大旗帜，全面贯彻党的十八大和十八届三中、四中、五中全会精神，以马克思列宁主义、毛泽东思想、邓小平理论、“三个代表”重要思想、科学发展观为指导，深入贯彻落实习近平总书记系列重要讲话特别是视察江苏重要讲话精神，坚持“四个全面”战略布局，坚持发展第一要务，牢固树立“五大发展理念”，以“迈上新台阶、建设新扬州”为主题，以全面提高发展质量和效益为中心，统筹推进经济建设、政治建设、文化建设、社会建设、生态文明建设和党的建设，加快形成引领经济发展新常态的体制机制和发展方式，加快构筑跨江融合发展新优势，着力建设经济强、百姓富、环境美、社会文明程度高的新扬州，率先全面建成小康社会，积极探索开启基本实现现代化建设新征程，奋力谱写好中国梦的扬州篇章。

“十三五”时期扬州经济社会发展的主要目标是：

——经济综合实力显著增强。地区生产总值年均增长9%左右，人均GDP稳定超省均。先进制造业和现代服务业成为现代产业体系的主干部分，农业现代化走在全省前列。新兴产业、高新技术产业产值分别占规上工业产值比重45%、48%，服务业增加值占GDP比重50%。“新产业、新人才、新城市”互动并进，基本建成区域性先进制造业基地和产业科技创新中心。大力实施产业、技术、模式、金融资本等创新，主要创新指标达到或超省均。全市各类人才总量达到100万。基本建成深度融入国家高速交通主干网的现代综合交通运输体系。县域经济加快崛起，城镇化建设水平保持苏中苏北领先地位。

——百姓富裕程度显著提升。按照“七个更”要求进一步提升民生保障水平。“三宜三业”城市建设取得新突破。社会就业更加充分，推进劳动报酬增长与劳动生产率同步提高。城乡居民收入增速高于经济增速，宝应、高邮、仪征城镇居民收入增速高于全市平均水平；城乡居民收入提前实现比2010年翻一番目标，力争全面达省均。社会保障制度更加公平更可持续。基础教育更加均衡，到2020年全面实现教育现代化。打造“健康中国”的扬州样本，现代医疗卫生体系建设取得重要进展，居民健康主要指标达到国际先进水平，体育人口比例达40%。市民居住品质进一步改善，舒适宜居的人居环境显著提升。

——生态环境质量显著改善。以生态文明建设倒逼转型发展，实现物质与生态“两个财富”同步积累。推进绿色生产、生活和消费，国家生态文明建设示范市、全国水生态文明建设试点城市建设取得重要成果，积极申办世界园艺博览会。地表水好于Ⅲ类水质比例73%以上，空气优良天数比例≥72%。大力发展生态循环经济、绿色低碳产业，资源开发利用效率大幅提高，主要污染物排放量大幅减少，环境风险得到有效控制。主体功能区和生态安全屏障基本形成，公园体系基本建成，城乡生态环境显著改善，生态文明制度体系更加健全。

——社会文明程度显著提高。中国梦和社会主义核心价值观更加深入人心，“勤奋勤勉、开拓进取、包容通融、崇文尚德”的城市精神广泛弘扬。人民群众思想道德素质、科学文化素质、健康文明素质全面提高，全社会法治意识不断增强。名人名品名作成果丰硕，“扬州好人”效应进一步彰显，公共文化服务体系更加完善，文化体制改革取得重要突破，文化软实力影响力持续扩大，国际文化旅游名城建设取得重大进展。文化产业成为支柱产业，增加值占GDP比重7%。

——各方面体制机制更加完善。城市治理体系和治理能力现代化取得显著成效，重要领域和关键环节改革取得决定性成果，各方面制度更加成熟定型。加快融入国家“一带一路”、长江经济带和长三角一体化建设，跨江融合发展取得更大突破。营造法治化国际化便利化市场环境，发展更高层次开放型经济。法治扬州建设扎实推进，人民民主更加健全，法治政府基本建成，司法公信力明显提高，人权得到切实保障，产权得到有效保护。法治建设满意度、公众安全感分别达90%、92%。

2016年工作安排

2016年，我们将深入贯彻落实党中央提出的创新、协调、绿色、开放、共享发展理念和去产能、去库存、去杆杠、降成本、补短板等供给侧结构性改革重点任务，按照市委确定的“稳中求进、创新发展”总基调和“重大产业项目建设优化年”、“美丽宜居城市建设年”、“公务管理规范年”相关要求，围绕扬优势、补短板、求突破，进一步放大扬州“生态、文化”特色和竞争力，进一步推进融合发展、更高水平开放、现代服务业提质增效、城市品质提升、县域经济壮大、城乡居民增收等重点工作，以新要求、新站位、新作为，牢牢把握加快科学发展的主动权。

全市经济社会发展主要预期目标是：地区生产总值增长9%，一般公共预算收入增长9%，居民消费价格指数不高于省定目标，城镇登记失业率控制在3.5%以内，全社会研发投入占GDP比重2.4%左右，城镇、农村常住居民人均可支配收入增长力争快于GDP增长，完成节能减排省定目标。

突出抓好以下八个方面工作：

1.深入实施创新驱动战略，着力提升发展质量和效益

主动把握和积极适应新常态，加快转型升级，扩大有效投入，推动经济中高速增长、中高端发展。

促进重大项目提质发展。编制产业项目建设规划，强化项目绩效评价，突出投资强度、地区总部经济、科技创新、列省重大项目等导向性考核。重点瞄准世界500强和行业100强，主攻“中国制造2025”、“互联网+”和新兴产业项目，提升项目建设的质量和效益。新开工重大工业项目50个，其中基本产业项目60%以上、智能化和绿色化提升项目40%；新开工重大服务业项目40个，其中现代服务业和旅游业项目60%以上。新开工、新竣工重大农业项目各22个。实施亿元以上重大科技成果转化项目50项；重点技改项目150项，工业技改投入增长13%。

加快推进科技创新。支持市经济技术开发区、扬州高新区加快发展。推进智能装备产业园、生物健康产业园、军民融合产业园等“多园同建”。推动生态科技新城、高邮湖西新区等创建省级高新区。每个县（市、区）建设1-2个科技创新中心。实施“2020高技术领航行动计划”，开展100项产业关键共性技术攻关。启动新一轮“科教合作新长征”计划，建立健全产业技术创新国际合作机制。签订产学研合作协议400项。实施“上市、专利、品牌、标准”战略。培育国家高新技术企业60家、省级以上“两站三中心”30家。授权发明和实用新型专利6000件。创省级以上品牌30个。“全国质量强市示范城市”创建通过国家级验收。引进高层次领军人才100名、专业技术人才1000名。

大力发展先进制造业。以“智能、绿色、品牌、服务”为方向，走“专精特新”之路。汽车产业重点提升关键零部件效益和集群效应，发展新能源汽车，做大汽车后市场，加快建设上海大众30万台发动机等一批零部件重大项目。机械产业重点在智能化、成套化和后续服务增值上形成新优势，智能装备产销突破300亿元。优化整合船舶产业，培育高技术特种船舶和海洋工程装备，发展船舶配套产业。促进化工园区绿色发展、链式发展。以新技术、新业态、新模式为着力点，构建战略性新兴产业发展体系，推动新能源、半导体照明、新材料、智能电网、节能环保产业提档升级，重点突破高端装备制造、新一代信息技术、生物技术和新医药产业。战略性新兴产业增加值占GDP比重达16%。

提升发展现代服务业。以“一基地三板块”为重点，加快上海（扬州）网络视听产业园落地运营和扬州软件园规划建设，推动江苏信息服务产业基地向数据服务、平台运营等领域转型。全年软件和信息服务业业务收入增长30%。科技服务业重点聚焦技术研发、检验检测、创业孵化等领域，加快在人才、机构、业态、集聚区方面形成规模。建设辐射周边的产业技术研究院和技术交易市场。新引进银行机构1-2家，支持设立各类天使基金、创投机构。完成直接融资200亿元。新增“新三板”挂牌企业20家。推动传统商贸转型发展，培育年交易额突破百亿的批发市场2个以上。加快空港新城及航空物流、扬州港“亿吨大港、百万标箱”建设。服务业增加值占GDP比重达45%。

2.持续深化改革开放，进一步激发发展内生动力

坚持内外需协调、高水平引进来走出去并重、优化企业发展环境与激发企业创新活力并举，不断增创发展新优势。

继续深化重点领域改革。深入推进跨江融合发展综合改革试点，加快宁镇扬同城化步伐，在科教、产业、交通、环境、旅游等方面开展专项对接。推进涉审中介机构改革，打造涉审中介服务网上超市。加强供给侧结构性改革，提高供给体系质量和效率。引导非国有资本参与国企改革。深化市场要素配置改革，促进人才、资金、科研成果在不同区域、所有制间有序流动。构建现代财政制度，推动事权与支出责任相适应的制度建设。深化供销合作社综合改革，打造服务“三农”的综合平台。

切实优化投资消费结构。创新重点领域投融资机制，带动社会资本投向新兴产业、公共服务、生态建设、基础设施等领域。全社会固定资产投资增长15%。扩大健康、养老、旅游、文体等服务消费，引导消费向智能、绿色方向转变。鼓励商贸企业创新商业模式，加强与知名电商平台合作，电子商务交易额增长30%。优化商业综合体布局规划，打造提升一批特色商业街区。社会消费品零售总额增长9%。建立购租并举的住房制度，多渠道发展房屋租赁市场，扩大刚需，化解库存，稳定房地产市场。支持建筑业转型升级，建筑业总产值达3350亿元。

进一步优化企业发展环境。优化涉企审批服务。进一步正税清费，合理降低企业综合税负水平。强化执法行为监管、服务创新和责任追究。实施信贷投放倍增、直接融资提升、优化金融生态等工程，引导金融资本助推实体经济。用好“创新券”、“技改券”，支持企业提升全要素生产率。深入推动“大众创业、万众创新”，启动国家小微企业创业创新示范城市创建工作。开工建设科技产业综合体和众创空间100万平方米，创成省级以上科技企业孵化器12家，入驻企业1000家，注册创客数3000人。个体工商户、私营企业数净增15000户、8000户以上，“个转企”1600户以上。

不断提高对外开放水平。实施新一轮“530”招商行动计划，引进世界500强和跨国公司项目6个以上，实际利用外资10亿美元。做好城市公共外交，深化与“一带一路”沿线国家的科技、经贸、文化、旅游合作。启动跨境贸易电子商务服务试点城市创建。落实展会补贴、电子口岸功能拓展、通关便利化等措施，支持企业“走出去”。高新技术产品出口比重超过15%。全年外经营业额增长10%。深化与上海、苏南园区合作共建，各省级以上开发区分别落户亿元以上项目3个以上。推进“中意（扬州）食品工业园”等中外合作园区建设。支持市经济技术开发区创成国家综合保税区，江都经济开发区争创国家级开发区。

3.加强“三农”工作，加快城乡一体化进程

积极走增产增效并重、生产生态协调、强农富农同步的发展道路，加快形成以工促农、以城带乡、城乡一体的新格局。

着力构建现代农业产业体系。加快国家农业科技园区和农业标准化示范区建设。实施粮食绿色增产和水产生态健康养殖工程，新增高标准农田15万亩、设施农（渔）业12万亩。培育100个农村示范合作社，新建省级示范家庭农场20个。推进粮食生产全程机械化，改扩建区域性粮食烘干中心40个，新增粮食仓容10万吨。新型农业经营主体规模经营比重达54%。农业龙头企业销售收入增长9%。创新农产品电子商务等交易方式。推动农产品市场信息化网络村级全覆盖。做好第三次全国农业普查工作。

进一步抓好农产品食品质量安全。开展市区蔬菜、水果、水产品、畜禽四大农产品批发市场“净源行动”。新申报“三品一标”产品80个，质量认证产品占食用农产品比重40%。推进农产品联网检测系统乡镇全覆盖，市级以上农产品质量抽检合格率98%。建立食品电子追溯系统，建设市级食品药品检测中心。加大农副产品深加工、技术研发及销售模式创新，带动农产品销售业发展，食品产业实现产值300亿元。

推进新型城镇化健康发展。实施户籍制度改革，全面推行居住证制度。出台乡镇差别化发展指导意见，统筹推进市县镇村“四级”联动发展，做强做优县城镇，加大重点中心镇建设力度，规划建设一批特色小镇。制定全市城镇化试点工作实施计划，推进高邮市和月塘、邵伯、氾水镇国家新型城镇化综合试点，抓好省级试点政策的对接争取和落实。完成18个镇67个村环境综合整治，打造10个体现地域特色的“美丽乡村”。

继续做大做强县域经济。支持创建一批生态型、创新型的产业园区。整合提升一批工业集中区，推动产业链式化、特色化、专业化发展。引进和培育一批先进制造业的龙头企业。在项目建设、基础设施、公共服务等方面争取上级更大支持。加快推进国省干线公路建设，建成611省道高邮段及向阳河特大桥、352省道江都先导段，开工建设331省道宝应先导段和333省道高邮东段先导段，积极推进沪陕高速设立枣林湾互通。打通偏远乡镇对外通道，推进沿河地区和仪征丘陵山区加快发展。全面提升“四有一责”建设水平，沿江、沿河地区村集体经营性收入分别实现50万元、40万元。

4.推进生态文明建设，努力打造美丽扬州

实施生态文明建设工程，推动形成绿色生产方式和生活方式，全面打造自然环境之美、景观风貌之美、城乡协调之美。

加快建设主体功能区。按照优化、重点、限制和禁止开发四类主体功能定位，落实空间用途管制，调整优化全市生态红线保护规划。规划建设江淮生态大走廊和文昌路西延生态廊道。按序时推进宝应湖、高邮清水潭、“七河八岛”等生态中心建设。强化生态文明制度体系建设，出台规范性文件，加强部门合作联动，提升执法水平。

着力推动绿色生产。切实降低能源、资源消耗，推进国土空间开发保护。开展建设用地总量和强度“双控”行动。围绕化工、建材等重点行业，推广关键共性清洁生产技术，完成60家企业清洁生产审核。鼓励支持战略性新兴产业、环保产业发展。加快园区循环化改造，维扬、高邮经济开发区分别创成国家和省级生态工业园。支持扬州（方巷）建筑产业园建设。开展绿色建筑行动，城镇新建建筑全部按一星及以上标准设计建造。全市12层以下住宅建筑全面推广应用可再生能源。

狠抓大气、水、土壤等重点环境治理。加强对工业废气、施工扬尘、机动车尾气等综合整治。强化燃煤污染治理，全部完成1100多台小型燃煤锅炉整治。构建完善的秸秆收贮体系，秸秆综合利用率达94%。实施重点断面达标保障和流域水污染治理行动，完成蔷薇河、向阳河等区域骨干河道整治工程。扎实推进“清水活水”工程，完成沙施河、七里河等综合整治。推进全国水生态文明建设试点市建设。实施土壤污染修复治理工程，加强农产品产地土壤重金属污染防治工作。

积极推进城乡生态建设。突出抓好“五路一环一河”和城市重要节点绿化提升工作，全市成片造林1.5万亩，市区新增城市绿地100万平方米。继续推进“绿色村庄秀美家园行动”。新建省级绿化示范村40个。恢复湿地4000亩，新建省级以上湿地公园2个。推进种植业控药控肥和养殖业减量减排，净化农产品产地环境。推行农业废弃物资源化利用，推广以沼气为纽带、种养结合的生态循环农业模式，新建农村沼气工程20个。

5.加快名城建设，推动城市功能形象新跃升

以获得联合国人居奖10周年为新起点，落实中央城市工作会议精神，持续提升城市品质，不断彰显历史文化名城魅力。

全力推进重大基础设施建设。加快推进连淮扬镇铁路和宿扬高速、江广高速公路扩容改造工程，开工建设东部客运枢纽。加快建设金湾路、城市南部快速通道，建成邗江南路、开发路东延等工程。全力做好城市轨道交通、京沪高速公路扩容及南延等项目前期工作。完成扬州泰州机场国际货运区改造、芒稻船闸扩容改造工程。加快华电扬州天然气发电、扬州港扩容等项目建设进度。基本建成一流配电网。

进一步完善城市功能和布局。加快改善老城区生活条件，推进消防、救护、避灾等通道和公共空间建设。围绕产城融合，提升西区新城、广陵新城、生态科技新城建设水平。推进新大剧院、球类活动中心等公共配套服务设施建设。科学规划建设城市公园体系，建成市级公园3座、区级公园2座、社区公园31座。完善市政管网系统。促进“海绵城市”建设。提升城市供水保障能力和运行质态，加大供水水质监管力度。构建高效安全的燃气应急保障机制，推进应急储备气源建设。

切实提升城市精细化管理水平。开展“七强化一提升”行动，全面整治市区流动摊点、出店经营，主城区流动摊点总数减少30%。集中整治1-2条餐饮街区，城市主干道、居民小区全面取缔露天烧烤。全面实施中心城区环卫设施布局规划。

运营好生活垃圾焚烧发电厂、餐厨废弃物处理厂等项目，生活垃圾无害化处理率95%以上、餐厨废弃物处理率30%以上。新改建垃圾中转站3座、公厕76座。全程化推进垃圾分类处理。道路清扫保洁机扫率88%以上。完成高层建筑专业救援消防执勤楼建设，加快建设电梯应急处置服务平台。

着力打造国际文化旅游名城。推动文化与旅游、科技等融合发展。加快扬州城遗址公园和隋炀帝墓遗址公园建设。提升“文化博览城”建设水平。参与“海上丝绸之路”联合申遗和丝路国际艺术节、文化之旅等活动。文化产业增加值占GDP比重达5%。制定旅游国际化三年行动计划。放大“世界运河历史文化城市合作组织”影响力，打造“中国国际运河文化旅游目的地”品牌。推进市县联动，每个县（市、区）打造1-2个重点旅游项目，启动建设10个特色旅游名镇。东关街争创国家5A级景区。完善全市域智慧旅游服务体系。提高游客服务中心建设管理水平。争取纳入居民赴台个人旅游试点城市。旅游业增加值占GDP比重达7.5%。

6.坚持共建共享，不断增强人民群众获得感和满意度

坚持普惠性、保基本、均等化、可持续方向，扩大有效供给，创新提供方式，不断提高公共服务共建能力和共享水平。

优先促进居民增收。围绕高质量就业、创业带动就业，多渠道增加城乡居民收入。建立重点企业用工服务绿色通道。全市新增城镇就业5万人。全年创业培训4000人，扶持成功创业2000人。加大妇女创业就业免费培训力度。培育生产经营型、专业技能型和专业服务型农民1.5万人。转移农村劳动力就业1.1万人。开展“离校未就业高校毕业生就业促进计划”。有就业意愿的困难家庭毕业生就业服务率100%。加强城乡就业困难人员“一对一”帮扶，确保城镇零就业和农村零转移家庭动态清零。

稳步提高社会保障水平。实施全民参保登记计划，加快推进社会保险全覆盖。全面落实新农合大病保险制度和职工大病补充保险制度。推动工伤预防、补偿、康复“三位一体”的保险服务体系建设。城乡基本养老、城镇基本医疗保险均新增1万人，工伤、生育保险分别新增3万人、2万人。完善申请救助家庭经济状况核对机制，健全困境儿童、残疾人急难救助分类保障制度。鼓励社会力量进入养老产业，提供多元化为老服务。

扎实做好重点民生工作。深化“不淹不涝”城市建设，启动瓜洲外排泵站建设。开通主城区经仪征天乐湖至六合客运班线，新辟、调整公交线路12条，新开通3个乡镇镇村公交。加强市区潮汐拥堵治理，实施道口改造，推行智能交通引导。规划建设市区智能停车运营管理系统，新改建公共停车场5个。新改造农村危房1260户、渔民上岸安居1432户。新改建农村道路192公里、危桥465座。疏浚县乡骨干河道80条。取缔马路市场20处，完成建成区57个农贸市场整治，新改建市区农贸市场12个。启动新一轮“八老”改造工程。解决市区超腾仓期未安置住房9729套。全市护理型床位占养老床位总数35%以上。实施新一轮扶贫开发工程，25%建档立卡低收入农户实现脱贫。

大力发展社会事业。推进教育现代化建设，新改建公办幼儿园6所，新创省义务教育现代化学校50所。推进职教集团与扬州产业深度融合，创新企业、政府、学校共建实训基地新模式，中职毕业生就业率98%以上。落实与扬州大学全面合作协议。推进扬子津科教园区和江苏旅游职业学院建设。启动规划扬州技师学院迁建，提标建设扬州老年大学。建成东部市民图书馆。承办第六届“江苏书展”。启动实施“健康扬州”工程。推进医疗、医药、医保联动，深化公立医院管理体制和运行机制改革。加快全市医联体建设，大力推行分级诊疗制度。开展12类45项基本公共卫生服务。继续办好扬州鉴真国际半程马拉松赛，提升赛事的品质和内涵。积极筹备第十九届省运会，加快体育场馆建设。举办首届市社区艺术节，办好全民健身体育节。

7.创新社会治理，切实提高社会文明程度

践行社会主义核心价值观，坚持系统治理、依法治理，维护社会公平正义，推动物质文明和精神文明协调发展。

不断提升公民文明素质和法治意识。持续实施文明城市建设常态化长效化三年行动计划。弘扬社会公德、职业道德、家庭美德，引导广大市民争做“扬州好人”。深入实施全民科学素质行动计划。加强社会诚信体系建设，推进信用信息互联互通和交换共享。启动实施“七五”普法规划。建成市公共法律服务中心，实现社区律师工作室全覆盖。推进建立公职律师制度。市区免费提供法律援助2000件以上。

加强基层基础工作。探索社区减负增效的办法措施，全面落实“社区盖章”目录制和准入制。大力发展公益性、服务性、互助性的社区社会组织，编制购买服务目录与具有承接能力的社会组织目录。推进科技强警，完善全市域公共安全视频监控系统，加强基层派出所建设。创新流动人口管理模式，进一步扩大向居住证持有人提供公共服务的范围。

保障群众利益诉求。健全群众利益表达、利益协调、利益保护机制。落实信访首办责任，完善联合接访工作。严格实行诉讼与信访分离，把涉法涉诉信访问题导入司法渠道解决。建立律师代理涉法涉诉信访案件制度。完善矛盾纠纷网格化排查预警机制。健全人民调解组织网络和工作机制，推进专业性行业性人民调解组织建设。加快市医患纠纷调处服务中心建设。

维护社会和谐稳定。深化“平安扬州”建设，以信息化为支撑加快建设现代化社会治安防控体系，重视做好反恐处突工作。实行重大决策社会稳定风险评估。切实做好社区矫正和安置帮教工作。继续保持“打非治违”高压态势，深化道路交通、消防、危险化学品、烟花爆竹等重点行业领域专项整治。加强应急体系建设。强化气象、地震基础设施建设，完善应对突发自然灾害救助体系和运行机制。支持国防和军队改革，深入实施军民融合发展战略，提升国防动员建设水平。

8.加强自身建设，打造高效清廉政府

坚持政府服务国际化和行政行为法治化，强化法治思维、法治方式，更大力度建设法治政府、阳光政府和服务型政府。

强化为民靠民，切实提升服务水平。坚持“民之所望、施政所向”，密切联系群众，尊重基层首创，继续深入开展“三下三联三交”活动，打通联系服务群众的“最后一公里”。深入基层和企业开展调查研究，切实解决企业和群众的实际困难与问题。加强数字化便民服务平台建设。继续办好《寄语市长》和12345政府服务热线，进一步提高办结率和满意率。

强化公开透明，大力推进依法行政。深入贯彻落实《法治政府建设实施纲要（2015-2020年）》。认真接受市人大的法律监督、工作监督和市政协的民主监督，充分听取各民主党派、工商联、无党派人士和社会各界的意见。切实做好人大代表议案、建议和政协提案办理工作。健全司法监督、审计监督、舆论监督制度和政府新闻发布制度。继续加强政务信息公开和互联网政务信息数据服务平台建设。健全政府立法程序规则，构建政府立法制规工作体系。坚持市政府常务会学法学纪制度。系统推进政府法律顾问工作。推行行政执法公开制度，严格规范公正文明执法。加强行政复议规范化建设。

强化严实作风，不断提高行政效能。深化“三严三实”专题教育，倡导“马上就办”精神，将“严”的作风和“实”的态度融入政府日常工作。继续简化办事手续，优化审批流程，推行“联合踏勘、联合审图、联合测绘、联合验收、区域性联合评价”等方式方法。加快制度创新，继续完善行政审批事项等清单管理制度。开展重大政策举措第三方评估，提高决策科学化水平。

强化崇俭戒奢，着力打造勤廉政府。落实廉洁自律主体责任，严格执行《准则》、《条例》和中央“八项规定”，驰而不息纠正“四风”。全部公开财政资金使用部门和单位的预决算及“三公”经费预决算。继续深化“三直接”十大环节操作规范，前延后伸需要规范的操作环节。自觉维护政府形象，以政务诚信带动社会诚信。加强勤政务实、敢于担当、清正廉洁的公务员队伍建设，为“十三五”规划的顺利实施提供有力支撑和保障。

各位代表！做好今年工作，实现“十三五”良好开局，使命崇高，责任重大。让我们紧密团结在以习近平同志为总书记的党中央周围，在省委省政府和中共扬州市委正确领导下，团结和依靠全市人民，同心同德，求真务实，锐意进取，为建设经济强、百姓富、环境美、社会文明程度高的新扬州而努力奋斗！
